# Amyloidosis & Advanced Heart Failure: When to Consider Cardiac Transplantation and What are the Results?

**DOI:** 10.1007/s11897-026-00777-6

**Published:** 2026-09-08

**Authors:** Steven Hamilton, Harsh Patolia, Mazen Hanna

**Affiliations:** Amyloidosis Center, Section of Heart Failure and Cardiac Transplant, Department of Cardiovascular Medicine, Cleveland Clinic, Cleveland, OH USA

**Keywords:** Cardiac amyloidosis, Transthyretin, Light chain, Advanced heart failure, Heart transplantation

## Abstract

**Purpose of Review:**

The management of end-stage heart failure in cardiac amyloidosis due to both light chain (AL-CA) and transthyretin (ATTR-CA) with heart transplantation has historically been limited by concerns about systemic progression after transplant. As a result, heart transplantation as a strategy was often restrictive and many patients were excluded. This review synthesizes contemporary evidence and outlines a practical framework to guide heart transplant evaluation and decision making in advanced disease. We emphasize evolving multidisciplinary selection principles.

**Recent Findings:**

In the recent era (since the 2010s), multicenter experience has demonstrated that carefully selected patients with advanced AL-CA and ATTR-CA can achieve favorable outcomes after heart transplantation. Specifically, selected individuals with wild type transthyretin cardiac amyloidosis (ATTRwt-CA) may be appropriate candidates. For hereditary transthyretin cardiac amyloidosis (ATTRv-CA), isolated heart or combined heart liver transplantation (CHLT) can been considered, although modern transthyretin targeted therapies increasingly influence this decision. Patients with AL-CA may undergo transplantation alongside timely plasma cell directed treatment.

**Summary:**

Historically, outcomes for heart transplantation in patients with cardiac amyloidosis were poor. Improved patient selection as well as advances in plasma cell targeted therapy for AL-CA and transthyretin stabilizing or gene silencing treatments for ATTR-CA have reshaped expectations. The improved outcomes seen in more contemporary cohorts support heart transplantation as a viable option for selected patients with advanced disease.

**Graphical Abstract:**

**Figure:**
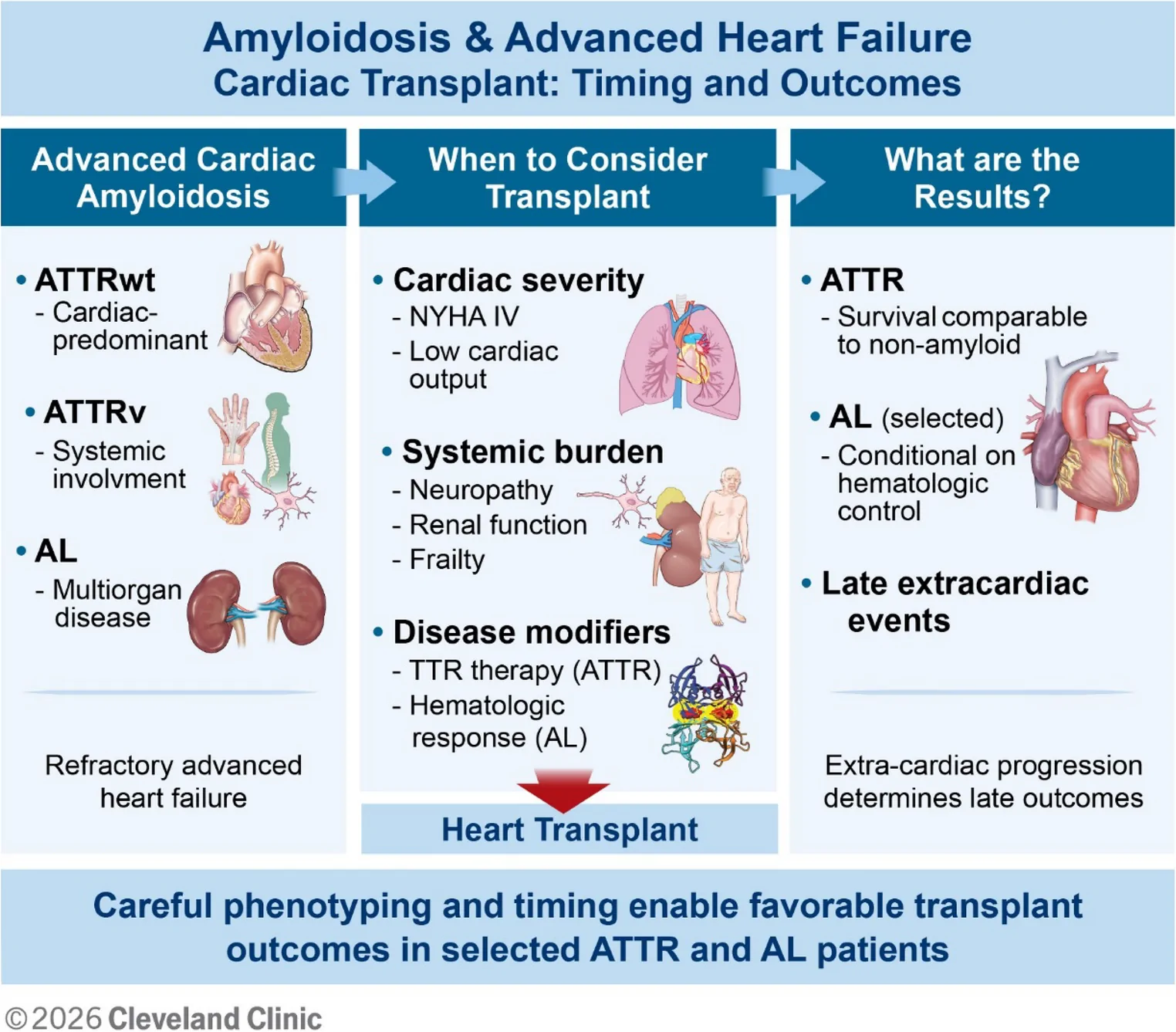

## Introduction

Cardiac amyloidosis (CA), either due to light chain (AL) or transthyretin (ATTR) amyloidosis, is characterized by the deposition of amyloid fibrils in the heart, which ultimately causes progressive heart failure (HF), arrhythmia, and/or electrical conduction disease. CA has become a more well-recognized etiology of heart failure (HF) due to increased awareness and better diagnostic imaging modalities, which has led to improvements in earlier diagnosis [1]. Additionally, the continued emergence of disease-modifying therapies has improved outcomes for both AL-CA and ATTR-CA, including better survival, reduced hospitalizations, and improved quality of life [2–5]. However, despite earlier diagnosis and emerging treatment options, patients with CA may occasionally be diagnosed at more advanced stages, may progress to end-stage HF despite treatment, or may present in cardiogenic shock. In these instances, advanced therapies such as heart transplantation (HT) represent a possible option in selected cases.

Over time, there has been an increase in HT for CA. This strategy has evolved from a controversial intervention to a viable treatment option, with survival now comparable to non-amyloid cardiomyopathies in appropriately chosen patients. In AL-CA, improvements in post-transplant outcomes reflect careful selection (particularly regarding the evaluation of extracardiac involvement) and the availability of more effective chemo/immunotherapy regimens (including stem cell transplantation) that can sustain hematologic remission. For ATTR-CA, recent registry data indicates robust post-transplant survival, with ongoing ATTR-directed therapies potentially offering additional post-transplant stabilization of extracardiac progression [6, 7].

**Fig. 1:**
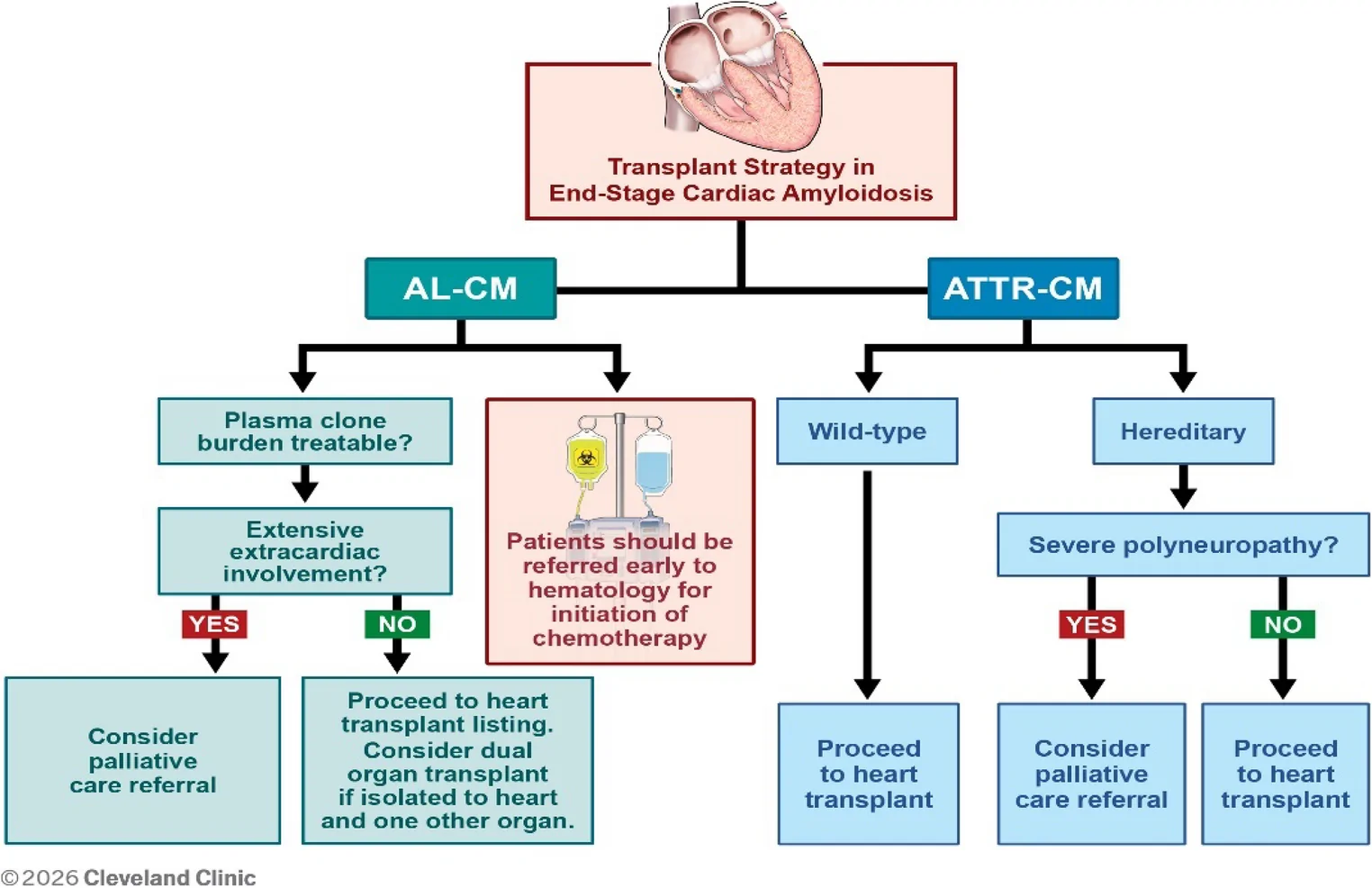
Subtype-specific framework for heart transplant candidacy. In AL-CA, chemotherapy initiation and extra-cardiac disease burden guide eligibility, with considerations of dual-organ transplantation in selected patients and palliative strategies when systemic involvement is extensive. In ATTR-CA, candidacy is stratified by wild-type versus hereditary disease and by severity of polyneuropathy. Heart transplant consideration is determined by amyloid subtype, systemic involvement, and functional status

The decision of when to consider HT and for whom remains nuanced and requires the integration of cardiac staging, an adequate assessment for extracardiac involvement and comorbidities, and the ability to treat the underlying disease after HT. A phenotype-directed framework for transplant decision-making in advanced cardiac amyloidosis is summarized in the central illustration (Fig. 1), highlighting key determinants of candidacy across AL and ATTR subtypes. This review delineates the features of advanced cardiac amyloidosis, reviews the criteria for transplant referral and candidacy, and summarizes the outcomes of HT across ATTR-CA and AL-CA subtypes. Given that ATTR-CA and AL-CA are two very disparate conditions, especially when it comes to considerations for advanced therapies, they will be discussed separately.

## Transthyretin Cardiac Amyloidosis (ATTR-CA)

### Introduction

ATTR-CA, due to the misfolding of the liver-derived protein transthyretin (TTR), is either due to wild-type disease associated with aging (ATTRwt-CA) or due to a hereditary variant (ATTRv-CA). There are over 150 variants in the TTR gene that cause hereditary ATTR amyloidosis, with the most common being p.Val142Ile mainly seen in African Americans. The amyloid fibrils in ATTR amyloidosis most commonly involve the heart, ligaments, and nerves, with nerve involvement being much more common in certain variants that cause ATTRv amyloidosis (i.e. p.Val50Met). Nerve involvement may lead to peripheral and/or autonomic neuropathy, and this can affect mobility, postural blood pressure, and thus be a concern for HT consideration, as will be discussed later. When ATTR-CA is diagnosed at an advanced stage of the disease or progresses to this stage, it is characterized by severe restrictive physiology, persistently elevated filling pressures and congestion, and end-organ dysfunction. For both ATTRwt-CA and ATTRv-CA, disease-modifying therapies (TTR stabilizers and gene silencers) have meaningfully improved outcomes [2–4], but they do not reliably reverse advanced myocardial infiltration once patients reach stage D heart failure, and HT may be considered. Notably, considerations for HT in patients with ATTRwt-CA may differ from those with ATTRv-CA, given more neurological involvement in the latter, as well as continued production of variant TTR by the liver post-HT.

### Staging and Markers of Advanced Disease in ATTR-CA

Defining and recognizing advanced ATTR-CA is similar to defining end-stage heart failure from other causes. Given ATTR-CA’s restrictive hemodynamics, patients may have only a mildly reduced ejection fraction yet have severe limitations attributable to fixed stroke volume, chronotropic incompetence, and right-sided failure. Patients with advanced disease have higher NT pro BNP (typically over 3000 ng/ml), lower estimated glomerular filtration rate (eGFR), require higher doses of diuretics, and have recurrent heart failure hospitalizations [8].

### HT Selection Considerations for ATTR-CA

When working up a patient with ATTR-CA for HT, assessment of the kidneys, lungs, liver, vasculature, pulmonary vascular resistance, and defining comorbidity burdens such as diabetes, frailty, and vascular disease remain standard. While ATTRwt-only datasets remain small, the available ATTRwt-focused and ATTRwt-enriched cohorts consistently indicate that isolated HT can deliver near-standard transplant survival in appropriately selected candidates, with excellent symptomatic recovery [7, 9, 10]. The 2024 ISHLT guideline for the care of heart transplant candidates explicitly recognizes improved outcomes in cardiac amyloidosis in the modern era and supports contemporary evaluation frameworks [11]. Given the difference between ATTRwt-CA and ATTRv-CA they will be discussed separately.

#### Variant Type ATTR-CA (ATTRv-CA)

Defining the specific TTR gene variant in an individual patient being considered for HT is important to predict the degree and trajectory of extracardiac involvement. The most common variant in the United States, p.Val142Ile, found mainly in African American patients, typically leads to a later onset of disease (median age around 70 years) with mainly cardiac involvement and usually mild neuropathy. As such, the neuropathic involvement in this variant is typically not as much an issue when it comes to candidacy for HT. Other variants, such as p.Thr80Ala, that can have more problematic peripheral and sensorimotor neuropathy may present a greater challenge with HT given the concern of progression post-HT leading to immobility, severe orthostasis, malnutrition, and diarrhea. Patients with more advanced peripheral and autonomic neuropathy may not be candidates, as even with concomitant liver transplant and/or disease-modifying therapies, including silencers, the neuropathic symptoms may not improve [12–14].

#### Wild Type ATTR-CA (ATTRwt-CA)

Patients with wild-type ATTR are typically older and present with predominantly cardiac involvement; as such concerns for extracardiac involvement that would limit HT outcomes are not as relevant per se. In ATTRwt amyloidosis, peripheral neuropathy tends to be milder, and these patients rarely have autonomic dysfunction significant enough to lead to problematic orthostatic hypotension (as compared to some variant forms of ATTRv amyloidosis). ATTRwt amyloidosis does not clinically affect the kidney; rather, renal dysfunction is related to cardiorenal physiology or other unrelated intrinsic factors that the patient may have.

### Extracardiac Disease

Careful assessment of extracardiac ATTR amyloid burden is central to contemporary HT evaluation, reflecting the recognition that ATTR amyloidosis is a systemic proteinopathy rather than an isolated cardiomyopathy (Table 1). Modern selection frameworks, therefore, place substantial emphasis on detailed neurologic (polyneuropathy, autonomic dysfunction, orthostatic intolerance), renal [proteinuria and estimated glomerular filtration rate (eGFR) trajectory], and gastrointestinal or nutritional assessments [13, 14]. In practical terms, extracardiac assessment is less about proving amyloid deposition in each organ and more about quantifying its clinical impact (trajectory, reversibility, and expected progression under contemporary ATTR-directed therapy) [11].

**Table 1:** Extracardiac manifestations of ATTR-CA and recommended pre-heart transplant appraisal

Extracardiac organ system	Clinical manifestation	Relevance to HT candidacy	Diagnostic appraisal
Gastrointestinal tract	• Early satiety • Diarrhea/constipation • Weight loss	• Malnutrition and sarcopenia increase waitlist time	• GI evaluation as indicated • Nutritional assessment • Serum albumin/prealbumin • BMI and body composition
Autonomic nervous system	• Orthostatic hypotension • Syncope • GI dysmotility • Erectile dysfunction, bladder dysfunction	• Predicts peri-operative risk, post-HT hypotension, and prolonged rehabilitation	• Orthostatic vitals • Medication review • Autonomic testing • Review of BP tolerance
Peripheral nervous system	• Length-dependent sensorimotor polyneuropathy (more common in ATTRv) • Pain, weakness • Gait instability	• Major determinant of post HT • Functional recovery, frailty, and quality of life	• Neurology consultation • Detailed neurologic exam • EMG/NCS if unclear phenotype • Functional assessments (gait speed, balance, ADLs)

### Integrating Extracardiac Findings into Candidacy: A Practical Synthesis

The impact of extracardiac assessment on risk for heart transplant should answer four questions:

1. Is extracardiac disease mild enough to allow meaningful rehabilitation and durable independence?
2. Is the trajectory stable or modifiable with contemporary ATTR therapies?
3. Are there organ-specific features that mandate a combined transplant strategy (heart–kidney; heart–liver), or that represent a contraindication? [15, 16]
4. What is the plan for post-HT surveillance and systemic disease management? Recurrence of amyloid in the cardiac allograft appears uncommon in many series, but surveillance and systemic management remain clinically relevant, particularly as post-HT ATTR-directed therapy becomes more common [10, 17].

### Outcomes in ATTRwt

Contemporary series suggest that carefully selected ATTRwt patients can achieve post–HT survival comparable to non-amyloid transplant recipients, with excellent functional recovery in most survivors [13]. The earliest dedicated ATTRwt HT cohort reports excellent mid-term survival and objective functional improvement after isolated HT. In that experience, 3-year survival was 100%, with one non-cardiac death (pancreatic cancer) at 45 months post-transplant; peak VO and 6-minute walk distance improved substantially after HT. Notably, systemic manifestations (late GI and peripheral nerve involvement) emerged in some patients, underscoring that transplantation treats the cardiac end-organ but not the underlying systemic proteinopathy [9]. In carefully selected ATTRwt patients, isolated HT can deliver excellent cardiac and survival outcomes, but extracardiac ATTRwt manifestations may emerge over time.

Subsequently, a multicenter (UK, Italy) tertiary experience enriched for ATTRwt, including 11 ATTRwt and 3 ATTRv recipients (combined heart/liver excluded) provided the longest follow-up among modern ATTR-CA HT reports. Post-HT follow-up was substantial (median 66 months), and survival was excellent: 1-year 100%, 3-year 92%, 5-year 90%, with most surviving patients reporting NYHA class I functional status at censor [7]. This is one of the strongest datasets supporting isolated HT for ATTRwt, with systematic monitoring suggesting low observed risk of allograft recurrence through medium-to-long follow-up.

### Outcomes in ATTRv-CA

#### Outcomes of Isolated HT in ATTRv-CA

In patients with ATTRv-CA with certain variants such as Val122Ile who have a predominant cardiac phenotype and undergo isolated HT without concomitant liver transplant, clinically significant recurrence of amyloid within the cardiac allograft appears uncommon in available follow-up (Table 2). While isolated cases of cardiac allograft amyloid deposition have been described, recurrence has not emerged as a dominant mechanism of graft failure. Instead, late morbidity and mortality in ATTRv recipients are more closely linked to progression of extracardiac amyloid involvement, including peripheral and autonomic neuropathy, gastrointestinal dysfunction,, and frailty.

**Table 2:** Outcomes of heart transplantation in hereditary (ATTRv) and wild-type (ATTRwt) transthyretin amyloidosis

Study/year	Center/Design	ATTRv patients (*n*)	ATTRwt patients (*n*)	Median follow-up	Survival outcomes	Cardiac allograft recurrence	Extra-cardiac progression
Davis et al., [18]	Single-center	7	2	~ 12 months	1-year - 74%; no early excess mortality.	Rare (1 recurrence in the entire cohort)	Not systematically reported
Kristen et al., [45]	Single center, 2002-2007 (Era 1), 2008-2017 (Era 2)	16		Up to 10 years	5-year - 50% in Era 1, 75% in Era 2; comparable to non-amyloid	Not emphasized	Limited by strict selection
Rosenbaum et al., [9]	Single center, 2007 - 2015		7	Up to ~ 45 months	3-year - 100%, 1 death (pancreatic cancer) at 45 months	Not reported	Neuropathy (4/7) GI (2/7)
Barrett et al., [37]	Single center, 2004 - 2017	18		~ 3 years	No difference in mortality vs. non-amyloid HT	Not reported	Not detailed
Griffin et al., [6]	Single center, 2001-2007 (Era 1), 2008-2018 (Era 2)	21		Up to 10 years	Era 1: 1-yr 100%, 3-yr 67%, 5-yr 67% Era 2: 1-, 3-, 5-yr 100%	Not reported	Not detailed
Vaidya et al., [36]	Single center, 2010-2018	38		3.2 years	3-year - 86%	None detected on EMBx	Minimal reported
Griffin et al., [10]	Single center, 2002-2019	7	5	4.0 years	Survival not primary endpoint	None	>50% developed GI, neurologic, or orthopedic ATTR manifestations
Guendouz et al., [54]	Single center, 2005-2018	6		4.2 years	1-yr 100%, 2-yr 100%	None	Progressive peripheral neuropathy
Razvi et al., [7]	Two centers, 1990-2020	3	11	66 months	1-yr 100%, 3-yr 92%, 5-yr ~90%	None	Peripheral neuropathy developed in some ATTRv; treated post-HT with patisiran
Lyle et al., [19]	Single center, 2007-2020	28	17	~ 10 years	ATTRv: 1-yr 93%, 3-yr 93%, 5-yr 89% ATTRwt: 1-yr 100%, 3 yr 94%, 5-yr 80%	Not reported	Not reported

The studies are predominantly isolated heart transplants, with the following including dual or triple organs. Davis 2015: 1 HT-KT, Rosenbaum 2018: 2 HT-KT, Lyle 2025: ATTRv 20 HT-LT and 2 HT-LT-KT. ATTRwt 2 HT-KT

*HT* heart transplant, *KT* kidney transplant, *LT* liver transplant

Most of the evidence base for transplantation in ATTRwt-CA remains retrospective, and a strategy of transplantation among select patients with end-stage heart failure can provide survival benefits comparable to those patients with advanced heart failure without amyloid heart disease

Despite encouraging results, evidence gaps remain substantial. Most published series pool ATTRv and ATTRwt phenotypes, limiting genotype-specific inference, and long-term data beyond 10 years are sparse [7, 10, 18, 19]. Furthermore, the optimal integration of post-transplant TTR-targeted therapies remains undefined, with current practice extrapolated from non-transplant populations. Prospective registries incorporating genotype, extracardiac staging, and post-transplant disease-modifying therapy will be essential to refine outcome prediction and optimize long-term care.

#### Isolated Heart Transplant Versus Combined Heart–Liver Transplant in ATTRv

Because variant TTR is predominantly produced by the liver, combined heart–liver transplantation (CHLT) has long been used to (i) replace the failing heart and (ii) reduce ongoing production of mutant TTR, theoretically limiting systemic progression. The most mutation-informed outcome data come from the Familial Amyloid Polyneuropathy World Transplant Registry analysis of non-Val30Met mutations, which evaluated liver transplant alone versus combined liver/heart transplant strategies. Survival varied markedly by genotype, but mutations with such as p.Thr80Ala and some others demonstrated better long-term survival when the heart and liver were both transplanted compared with liver transplant alone in; [20].

In practice, the decision between isolated HT and CHLT is increasingly individualized: many centers now consider isolated HT for ATTRv patients with manageable extracardiac trajectories. Of note, isolated HT for the most common variant, p.Val142Ile, is feasible with no significant recurrence in the allograft but extracardiac progression in the nerves can occur late post-HT [21, 22]. At our center, HT without concomitant liver transplant is done in these situations and post-HT, TTR therapy is implemented to reduce the risk of extracardiac progression.

## Post–Heart Transplant Considerations for Treatment

Disease-modifying therapy is increasingly considered after HT, particularly in ATTRv, to reduce ongoing production/misfolding of transthyretin and to preserve extracardiac function (e.g., stabilizers such as tafamidis and silencers such as patisiran/vutrisiran in non-transplant populations). However, data specifically guiding their use after HT is sparse and practice is largely drawn from studies in non-transplant populations; decisions will need to be individualized [13]. Given that there is data for polyneuropathy and not stabilizers for ATTRv neuropathy, one could extrapolate that this would preferred if the goal is to avoid progression of preexisting neuropathy. Due to the availability of TTR stabilizing and silencing therapies, the need for CHLT in ATTRv-CA is likely to become obsolete, particularly since there are reports that even with liver transplant, wild type ATTR deposition can occur due to “seeding” leading to progression of TTR amyloidosis [23].

## Light Chain Cardiac Amyloidosis (AL-CA)

### Introduction

Light chain amyloidosis (AL) occurs due to an abnormal clone of plasma cells that produce monoclonal serum free immunoglobulin light chains that are prone to misfolding. These abnormally folded light chain proteins aggregate into amyloid fibrils that deposit into organs causing dysfunction [24]. While extracardiac involvement may include the kidney (proteinuria/nephrotic syndrome), gastrointestinal tract (malabsorption or dysmotility), the liver (hepatomegaly), and the nerves (peripheral neuropathy, autonomic dysfunction), this rare and systemic disease presents with cardiomyopathy in over 75% of cases [25].

Median survival is dependent on the severity of cardiac involvement at the time of diagnosis and approximated to five years in modern cohorts [26, 27]. One of the most important tenets in this condition is early treatment given the fact that prefibrillar circulating monoclonal light chains are directly toxic to the myocytes in addition to the deposition of inert amyloid fibrils, hence the term an “infiltrative-toxic” disorder. The ultimate goal is to induce a complete hematologic response while supportively managing organ specific disease and treatment related toxicities [24]. Although there can be a cardiac response in many cases, in certain circumstances, the heart may be severely affected and not improve, or the patient may initially present in cardiogenic shock. It is in these cases where HT may be necessary and warranted if feasible. Herein, we discuss and describe the staging and treatment of AL-CA as well as the role of HT in its contemporary management.

### Staging AL Cardiac Amyloidosis

Prognosis in light chain amyloidosis is determined largely by the presence and severity of cardiac involvement. To this regard, cardiac biomarkers have powerful prognostic value and are central to AL staging frameworks [26, 28, 29]. These staging systems for AL amyloidosis are summarized in Table 3.

**Table 3:** Comparison of staging systems for AL amyloidosis

Staging system	Criteria	Stages (Median survival)
Mayo 2004 Staging System [28]	• NT-proBNP ≥ 332 pg/mL • TnT ≥ 0.035 ng/mL	I: 0 criteria (26.4 months) II: 1 criteria (10.5 months) III: 2 criteria (3.5 months) **European 2015 Modification** IIIa: 2 criteria with NT-proBNP < 8500 pg/mL (26 months) IIIb: 2 criteria with NT-proBNP > 8500 pg/mL (6 months)
Mayo 2012 Staging System [26]	• NT-proBNP ≥ 1800 pg/mL • TnT ≥ 0.025 ng/mL • dFLC ≥ 18 mg/dL	I: 0 criteria (not reached) II: 1 criteria (68.8 months) III: 2 criteria (16.7 months) IV: 3 criteria (6.7 months)
Boston University 2018 Staging System [29]	• BNP > 81 pg/mL • TnI ≥ 0.1 ng/mL	I: 0 criteria (not reached) II: 1 criteria (9.4 years) III: 2 criteria with BNP ≤ 700 pg/mL (4.3 years) IIIb: 2 criteria with BNP > 81 pg/mL (1 year)

Median survivals should not be compared between models (including between the European modification to the 2004 Mayo Model and the 2004 Mayo Model itself) due to heterogeneity in follow-up periods among cohorts

### End-Stage AL Cardiac Amyloidosis: Who Should be Referred for Heart Transplant?

While therapy in AL amyloidosis is systemically targeted towards eliminating the plasma cell clone and achieving both a hematologic and organ response, extensive cardiac involvement should prompt evaluation for heart transplantation (HT) [30]. There is a considerable degree of heterogeneity in the presentation of AL-CA, and the 2025 Mayo Clinic consensus statement on heart transplantation in cardiac amyloidosis classifies three phenotypes of AL-CA patients in whom HT may be considered: (A) patients with cardiogenic shock (CS) at the time of AL-CA diagnosis, (B) patients receiving targeted hematologic therapy who continue to develop worsening heart failure (HF) symptoms, and (C) patients who have received complete remission (CR) or very good partial remission (VGPR) but continue to experience worsening HF after one year of observation [19].

In the framework noted above, phenotype B and C patients with American College of Cardiology (ACC) stage D heart failure with refractory congestive symptoms and severe cardiac dysfunction should be considered for urgent referral to a transplant center [31]. Urgent referral to high volume transplant centers is warranted among patients with refractory symptoms despite optimized medical therapy, as well as those with Mayo Stage III disease or higher. When clinically risk stratifying these patients, LV chamber size and LVEF should be interpreted cautiously as the magnitude of cardiac dysfunction and restrictive cardiomyopathy may not be accurately reflected by those markers alone. Consideration of referral for heart transplantation is nuanced, with particular attention to severity and stage of symptoms and heart failure hospitalizations, diuretic resistance, elevation in natriuretic peptides and troponin biomarkers, and low-output state. For this reason, we recommend that AL-CA patients be evaluated comprehensively with imaging, biochemical, and clinical assessments.

All patients with confirmed AL-CA should be evaluated using the previously discussed staging criteria, and it is reasonable to refer patients with Mayo stage IIIB disease for HT in the absence of contraindications. Notably, these risk stratification models were developed before the era of improved chemotherapies (such as proteosome inhibitors and the anti-CD38 monoclonal antibody Daratumumab), which have demonstrated improvement in cardiac biomarkers and performance [32]. Thus, sole reliance on these biomarkers may lead to over-referral for HT and may serve as an adjunctive tool in the global assessment of a patient with advanced heart failure due to AL-CA [19].Invasive hemodynamic profiling and heart failure therapy can also elucidate the reversibility of end-organ dysfunction (such as congestive hepatopathy or cardiorenal syndrome) and optimize HT candidacy.

Patients with cardiogenic shock at the time of diagnosis of AL-CA (Mayo phenotype A) are of higher acuity and require more urgent and expedited HT evaluation. With temporary mechanical circulatory support (MCS) implanted, selected patients may need to be listed for HT and transplanted while chemotherapy is being initiated and even prior to the ability to assess the response to chemotherapy given the time limitation. Data supporting this strategy among patients with cardiogenic shock in AL-CA is limited, and urgent HT for patients too sick to tolerate chemotherapy has been advocated by the Mayo 2025 consensus statement [19, 33]. The main risk factor in this case is whether or not the plasma cell clone will be responsive to treatment (including stem cell transplant) post heart transplant. Now, with even more emerging therapies such as bispecific antibodies, the ability to take a chance upfront is more palatable given the increasing options to induce hematologic remission.

### Evaluating Extracardiac Amyloid Burden before Heart Transplantation

Consideration for HT specifically in AL-CA should include multisystem evaluation of the extent of extracardiac involvement (Table 4). In the early era, patients with extensive extracardiac or systemic involvement (including advanced amyloid nephropathy or gastrointestinal amyloid infiltration causing advanced malnutrition cachexia) had been typically excluded from transplant consideration however the extent of extracardiac involvement that has been deemed acceptable has varied by center [34].

**Table 4:** Summary of extracardiac manifestations of AL-CA and recommended pre-transplant workup

Extracardiac organ system	Clinical manifestation	Workup
Kidney	• Anasarca and edema • Hypoalbuminemia • Worsening renal function by creatinine or estimated glomerular filtration rate.	• 24-hour urine protein > 0.5 g/day with predominant albuminuria with exoneration of other causes of proteinuria such as diabetes.* • Renal biopsy with evidence of amyloid deposits.
Liver	• Elevated alkaline phosphatase over 1.5 times the limit of normal or hyperbilirubinemia.† • Hepatomegaly with liver span greater than 15 cm by radionuclide or computed tomography imaging.	• Liver biopsy with perivascular and sinusoidal deposits of amyloid and evidence of synthetic dysfunction.
Gastrointestinal tract	• Diarrhea • Dysmotility • Weight loss • Malnutrition	• Intestinal biopsy via endoscopy or colonoscopy demonstrating mucosal amyloid depositing. • Evaluation of markers of malabsorption such as fat-soluble vitamins and other nutrients.
Lung	• Diffuse interstitial lung disease on CT or CXR. ◦ Diffuse interstitial pulmonary amyloidosis. • Localized nodules or alveolar septal disease on CT. ◦ Nodular pulmonary amyloidosis ◦ Tracheobronchial amyloidosis	• Pulmonary function testing. • Transbronchial lung biopsy or video-assisted thoracoscopic biopsy demonstrating interstitial pulmonary amyloid deposits. • Pleural biopsy demonstrating direct infiltration with amyloid or pleural fluid studies consistent with exudative effusion.
Neurologic	• Sensorimotor neuropathy ◦ Dysesthesia ◦ Paresthesia ◦ Progressive sensory loss • Autonomic neuropathy ◦ Postural hypotension ◦ Bowel/bladder dysfunction ◦ Recurrent falls	• Electromyography • Nerve conduction study • Nutritional assessment • Frailty scoring with objective measurements such as Modified Fried frailty score.
Bone marrow	• M-spike on serum or urine immunofixation. • Elevated serum free kappa or lambda light chains.	• Bone marrow biopsy with cytogenetics (including FISH) and assessment of PMBC.

*Degree of proteinuria that is acceptable varies by center and can be up to 2 g/day

† Alkaline phosphatase > 1.5X upper limit of normal is an arbitrary threshold

There are no extracardiac organ-specific thresholds to safely proceed with HT but the general principle is that it should not limit post HT transplant outcomes substantially. The DANGER criteria (diarrhea, autonomic nervous system, nutritional status, gastrointestinal tract, elimination/renal function, respiratory tract) have been previously reported as a framework to assess extracardiac burden and exclude high-risk AL-CA patients from HT [33]. The 2021 American Society of Transplantation consensus statement on solid organ transplantation in melanoma and hematologic malignancies recommends that transplantation in light chain amyloidosis only be considered among candidates with therapeutic response to hematologic therapy, single organ involvement with amyloidosis, inadequate criteria for symptomatic myeloma, and acceptable candidacy for stem cell transplantation after organ transplantation [35]. In practice, however, these criteria do not accurately reflect contemporary transplant practices at all amyloid referral centers [36, 37]. An approach to assessing extracardiac burden of light chain disease is summarized in Table 1.

#### Bone Marrow Assessment of Plasma Cell Burden

A bone marrow biopsy (BMB) with cytogenetics with hematologic consultation should be obtained to risk stratify patients and assess plasma cell clone burden and identify high-risk cytogenetics or myeloma defining criteria. Bone marrow clonal plasma cell (BMPC) burden of 20% or greater is less favorable for HT given the concern of recurrence though not strictly contraindicated and may be less relevant with more modern chemotherapy/immunotherapy regimens for AL amyloidosis. Free kappa and lambda light chains should be monitored throughout the treatment period to assess response to targeted therapy [13, 19].

#### Gastrointestinal System

Organ amyloid deposition, especially in the gut, does not reliably predict organ involvement and thus is not a contraindication for HT [37–39]. GI dysfunction due to amyloid infiltration versus autonomic dysmotility can be challenging to differentiate. Intestinal biopsy may be warranted if there is diarrhea, dysmotility, and malnutrition, and significant intestinal involvement causing malabsorption can be a contraindication to HT [19, 39]. Though nutritional assessment with body mass index (BMI) or nutritional risk index are vital in transplant consideration, they are not necessarily reflective of the degree of GI involvement in light chain amyloidosis [38]. With respect to liver involvement, elevated alkaline phosphatase and hepatomegaly are the manifestations. There are no clear cutoffs regarding the degree of liver involvement and the risk for HT, but the presence of hepatomegaly (ALP) should prompt consideration of liver biopsy to assess for interstitial amyloid deposits and cirrhosis or advanced fibrosis [19, 39, 40].

#### Peripheral Nervous System

A thorough assessment of patient functional status can provide a surrogate for the severity of autonomic and peripheral neuropathy. Severe orthostatic hypotension requiring medications such as midodrine often preclude HT given the concern for post operative vasoplegia. Provider and patient questionnaires, such as the validated Composite Autonomic Symptom Score-31 (COMPASS 31) as well as formal assessments such as electromyography and nerve conduction studies, can delineate the extent of autonomic and peripheral neuropathy in AL-CM [13]. Formal neurology consultation may be warranted in severe cases with gait impairment or motor instability that could impede post-transplantation rehabilitation [19].

#### Kidneys

Among patients with renal involvement, careful assessment with serum and urine chemistries to assess renal function and the degree of proteinuria is crucial [41]. Proteinuria in AL amyloidosis is due to glomerular involvement and various protocols will exclude a patient’s candidacy for HT alone based on a certain threshold in of 24-hour urine albumin quantification (center specific and may range from approximately 500 mg to 2000 mg). Patients with significant proteinuria or with end-stage kidney disease on renal replacement therapy, advanced chronic kidney disease (with eGFR < 30 ml/min per 1.73 m [2]) without a reversible cause, and those with acute kidney injury (AKI) requiring RRT for six weeks may be considered for heart-kidney transplant (HKT) [19].

#### Lungs

Localized and diffuse lung involvement in light chain amyloidosis has been described and can manifest as nodular parenchymal or alveolar septal disease with largely nonspecific radiographic findings on cross-sectional imaging with computed tomography (CT). Rarely, patients with pulmonary amyloidosis may also present with exudative pleural effusions with pleural amyloid infiltration, as well as diffuse interstitial lung disease due to pulmonary amyloidosis. Confirmation of pulmonary amyloidosis may require evaluation with transbronchial lung biopsy or video-assisted thoracoscopic biopsy, as well as formal pulmonary function testing (PFT) before proceeding with heart transplant. Significant diffuse pulmonary involvement can restrict candidacy for HT [39, 42].

### Post-Transplant Outcomes in the Early Era

AL amyloidosis was previously considered a contraindication to HT due to high mortality and rapid recurrence in the transplanted heart in the early 1980 s up until the early 2000 s (Table 5) [43, 44]. However, subsequent cohorts in more modern eras showed better survival due to improved selection and treatment of the underlying AL amyloidosis.

**Table 5:** Outcomes of heart transplantation in AL amyloidosis

Study author	AL transplant cohort size (Location)	Findings
Early era		
Dubrey et al., [43]	17 United Kingdom National Transplant Database	1982-2002 • 1-year survival 59% • 5-year survival 38% • Inferior survival compared to non-amyloid HT patients in transplant database (*p*=0.013). • 1-, 2- & 5-year survival for those patients who received chemotherapy was 71%, 71%, & 36%, respectively. • 1-, 2-, & 5-year survival rates for those patients who received chemotherapy was 86%, 86%, and 64%, respectively.
Lacy et al., [44]	**11** Mayo Clinic, USA	**1994-2005** • 1- and 5-year survival rates were 82%, and 65%, respectively.
EARLY & MODERN ERA		
Gilstrap et al., [49]	**31** Massachusetts General Hospital, USA	**2000-2011** • Mortality hazard ratio of 4.7 (95% CI, 2.8–11.8, *p*<0.001) while on waiting list compared to non-amyloid patients. • No difference in 6-year survival compared to non-amyloid CM (*p*=0.34).
Kristen et al., [45]	**32** University of Heidelberg, Germany	**2002-2007** • 1-year survival 69%. • 2-year survival 56%. • Inferior survival compared to non-amyloid CM post-HTx outcomes (*p*=0.042). **2008-2017** • 1-year survival 85%. • 2-year survival 85%. • Noninferior survival compared to non-amyloid CM post-HTx outcomes (*p*=0.372).
MODERN ERA		
Trachtenberg et al., [34]	**16** (7 of which received ASCT after HT) Houston Methodist Hospital, USA	**2004-2018** • 1- and 5-year survival rates were 87.5% & 76.6%. • No difference in long-term survival compared to nonamyloid CM (*p*=0.3082).
Barrett et al., [37]	**13** Stanford University Medical Center & Kaiser Permanente Santa Clara Medical Center, USA	**2004-2017** • 8% 10-year mortality among patients with AL-CA.
Vaidya et al., [36]	**13** Cedars-Sinai Medical Center, California, USA	**2010-2018** • 1-year survival 91%
Patel et al., [46]	**31** Houston Methodist Hospital, USA	**2009-2023** • 1-, 3-, 5-, & 8-year survival rates were 87%, 83%, 73%, & 67%, respectively. • No difference in survival compared to non-amyloid CM (*p*=0.515) • AL-CA was not significantly associated with increased mortality when compared to non-amyloid patients (univariate Cox hazard ratio [HR] 1.53, 95% CI 0.83–2.82, *p* = 0.175 & multivariate Cox HR 1.43, 95% CI 0.76–2.72, *p* = 0.267)

While AL-CA was previously considered a strict a contraindication to transplantation, the paradigm of managing patients with advanced heart failure has shifted towards both early evaluation for heart transplant in parallel with initiation of targeted chemotherapy in collaboration with hematologists

### Post-Transplant Outcomes in the Modern Era

Modern treatment protocols favor early collaboration with hematologists and initiation of chemotherapy with a regimen of proteasome inhibition such as bortezomib, immunomodulatory and immunotherapies, and steroids [45]. These therapies can be administered before, in parallel to, or after HT listing evaluation. Post-transplant outcomes using this algorithm have been similar to patients who undergo HT for other indications, with Barrett et al. reporting acceptable outcomes among 13 patients with AL-CA that underwent HT at two large academic centers between 2004 and 2017 [37]. Similar outcomes were observed among 31 patients at Houston Methodist Hospital who underwent HT for AL-CA between 2009 and 2023, with 1-, 3-, 5-, and 8- year survival rates of 87%, 83%, 73%, and 67%, respectively. These outcomes were not significantly different from non-AL-CA HT patients [46].

AL-CA patients with advanced cardiac involvement may be unable to safely tolerate hematologic therapies, especially autologous stem cell transplantation (ASCT). In these cases, a strategy of early HT can stabilize cardiovascular status to facilitate safe delivery of hematologic therapy. Treatment with chemotherapy after HT has been demonstrated previously with favorable results [33, 47]. Varr et al. at the Stanford University Amyloid Center reported that this strategy of up-front HT followed by chemotherapy among six patients was successful, with five out of the original six patients alive up to 25 months after HT without evidence of recurrent cardiac amyloid deposition [47]. Durable biventricular mechanical circulatory support (MCS) has also been safely used in these patients as a bridge to HT, with one center reporting 1-year post-transplant survival of 83% from 2008 to 2018 among patients requiring MCS [48]. ASCT can be performed at least 6 to 12 months after HT [34, 36, 49]. The safety of this delayed strategy of ASCT has been demonstrated by Trachtenberg et al. at the Houston Methodist Hospital, in which post-HT survival after 1 year and 5 years was 87.5% and 76.%, respectively, similar to institutional outcomes for patients that underwent HT for other reasons [33, 34, 36, 50]. If patients can be adequately controlled with chemotherapy after HT, then ASCT may not be necessary. Thus, routine ASCT after cardiac transplantation may not be indicated and is no longer performed by some centers [36, 37]. With major advancements in chemotherapy and patient selection, AL-CA should no longer be perceived as a contraindication to HT but rather as a mandate for urgent, multi-disciplinary evaluation. Table 5 provides a summary of outcomes in the early and modern eras.

### *Multiorgan* Transplantation *in AL-CA*

Both advanced kidney and heart failure in light chain amyloidosis may warrant evaluation for simultaneous heart and kidney transplantation. Irreversible kidney disease or substantial persistent proteinuria despite optimization of serum free light chains and cardiorenal syndrome, as well as other non-amyloid-related conditions such as diabetes mellitus, should prompt early involvement of transplant nephrologists for consideration of concomitant kidney transplantation. A chronic severe reduction in eGFR (< 30 mL/min per 1.73 m [2]) or prolonged need for RRT has been associated with poor renal outcomes after solitary non-kidney transplant [19]. Patients with high levels of proteinuria (UPCR of 1 or higher) can indicate significant glomerular involvement, and the degree of interstitial fibrosis and tubular atrophy on biopsy can accurately assess the magnitude of irreversible kidney damage [51, 52]. Long-term data among heart-kidney transplant recipients remain limited, though small studies have demonstrated acceptable outcomes [46, 53]. In a cohort of 17 AL-CA patients at the French National Referral Center for Cardiac Amyloidosis between 2005 and 2018, 5 patients underwent heart-kidney transplantation with outcomes comparable to HT patients without AL-CA [54].

Significant hepatic involvement with advanced liver fibrosis or cirrhosis is associated with worse prognosis in AL-CA [38, 40]. While this degree of liver dysfunction can restrict candidate for solitary heart transplant, simultaneous heart and liver transplantation in AL-CA should be considered in the absence of other contraindications [19]. Combined heart and lung transplantation can be considered in very selective cases where significant lung disease has been demonstrated (from diffuse pulmonary amyloidosis or other causes). A single case report documented a successful combined heart-lung transplant in a 57-year old patient with AL-CA and mixed obstructive restrictive lung disease with a stable post-transplant 6 month course [55].

## Conclusion

HT in severe advanced cardiac amyloidosis, both for AL-CA and ATTR-CA, is feasible with reasonable outcomes in the more modern era. However, this requires careful patient selection and post-HT care that addresses the systemic nature of amyloidosis to prevent recurrence in the allograft, but more importantly, to reduce extracardiac progression that can lead to morbidity and mortality. We recommend evaluation at high-volume amyloid/HT centers employing multidisciplinary protocols.

### Practical Checklists

ATTR-CA referral triggers:

1. ATTR-CA NAC stage III disease (NT pro BNP > 3000 and eGFR < 45 ml/min.
2. Reduced LVEF and/or low cardiac output symptoms.

AL-CA phenotype staging:

1. At baseline, obtain NT‑proBNP, cardiac troponin (cTnT or hs‑cTnT, or cTnI) and the difference in involved and uninvolved free light chains (dFLC) before initiating therapy.

A.Risk factors: NT‑proBNP ≥ 1800 pg/mL, cTnT ≥ 0.025 ng/mL (or validated equivalents for cTnI/hs‑cTnT), dFLC ≥ 18 mg/dL.

2. Stage I: 0 factors; Stage II: 1 factor; Stage III: 2 factors; Stage IV: 3 factors.

### Durable Left Ventricular Assist Device use in CA patients

The use of durable left ventricular assist devices (LVADs) in patients with CA has historically been limited, largely because the underlying physiology is poorly suited to isolated left-sided mechanical support. Patients typically have small, hypertrophied ventricles with restrictive filling, and many have coexisting right ventricular dysfunction, which together increase the risk of inadequate LV preload and early right heart failure after LVAD implantation. In carefully selected patients with CA—particularly those with a relatively larger LV cavity and preserved or only mildly impaired RV function—continuous-flow LVAD support can be achieved with reasonable short-term outcomes, often as a bridge to transplantation [56, 57]Taken together, durable LVAD therapy in ATTR-CA is best considered a highly individualized strategy, reserved for select patients and typically employed as a bridge to transplantation in experienced centers, rather than a broadly applicable destination therapy.

### Clinical Translation & Future Directions

Although therapies for both light chain (AL) and transthyretin (ATTR) amyloidosis have dramatically improved, patients with cardiac amyloidosis (CA) are still diagnosed at or can progress to advanced stages of the disease, limiting survival. In the past, heart transplantation (HT) for both AL-CA and ATTR-CA was limited by concerns about systemic recurrence (including in the cardiac allograft) and by suboptimal survival outcomes. However, in the more recent era, HT for CA is associated with improved survival, comparable to that of patients receiving HT for traditional indications. This is due to better patient selection and the enhanced ability to treat the underlying systemic disease. Practical point-of-care guidance includes: (1) ATTRwt-CA: isolated HT for NYHA IV with NT-proBNP > 3,000 pg/mL despite TTR therapy; (2) ATTRv-CA (Val142Ile): isolated HT + post-HT silencer; (3) AL-CA Mayo IIIB phenotype B/C: multidisciplinary hematology/HT evaluation within 2 weeks. For AL-CA, patient selection centers around an appraisal of the degree of extracardiac disease burden that could limit HT outcomes, as well as the responsiveness of the plasma cell clone in consultation with the hematologic team. For ATTR-CA, patients with wild type typically have limited neuropathy and are candidates for HT with good outcomes but can have extracardiac TTR disease progression in the nerves and soft tissues. For patients with variant ATTR amyloidosis, combined heart-liver transplantation can be considered, although isolated HT is feasible in the most common variant p.Val142Ile, particularly now that we have TTR disease modifying therapies that can be given post HT. Prospective registries evaluating post-HT TTR silencer efficacy represent the next critical evidence gap. The optimal use and timing of stabilizers or gene silencers after transplantation for ATTR-CA remain largely guided by extrapolation rather than evidence.

## Key References

- Lyle MA, Rosenthal JL, Nativi Nicolau JN, et al. Heart Transplantation for Cardiac Amyloidosis: Mayo Clinic Consensus Statement. Mayo Clinic Proceedings. 2025;100(9):1578–1605. doi:https://doi.org/10.1016/j.mayocp.2025.05.009.
  - ○ This source specifically provides an evaluation and management strategy for transplantation among patienst with end-stage heart failure due to cardiac amyloidosis.
- Kittleson Michelle M, Ruberg Frederick L, Ambardekar Amrut V, et al. 2023 ACC Expert Consensus Decision Pathway on Comprehensive Multidisciplinary Care for the Patient With Cardiac Amyloidosis. *JACC*. 2023/03/21 2023;81(11):1076–1126. doi:https://doi.org/10.1016/j.jacc.2022.11.022.
  - ○ This guideline document provides a general framework in the evaluation and management of patients with advanced cardiac amyloidosis.
- Razvi Y, Porcari A, Di Nora C, et al. Cardiac transplantation in transthyretin amyloid cardiomyopathy: Outcomes from three decades of tertiary center experience. Original Research. Frontiers in Cardiovascular Medicine. 2023-January-19 2023;Volume 9–2022doi:https://doi.org/10.3389/fcvm.2022.1075806.
  - ○ This manuscript provides real-world, longitudinal evidence supporting heart transplantation in ATTR-CA, helps contextualize modern survival expectations, and underscores the importance of systemic disease assessment and evolving medical therapy in transplant decision-making.
- Punnoose LR, Siddiqi H, Rosenthal JL, Kittleson M, Witteles R, Alexander K. Implications of Extra-cardiac Disease in Patient Selection for Heart Transplantation: Considerations in Cardiac Amyloidosis. Cardiac Failure Review 2023;9:e01. 2023;doi:https://doi.org/10.15420/cfr.2022.24.
  - ○ This paper provides a foundational framwork for extending patient selection beyond cardiac metrics to integrating extra-cardiac disease burden and including comprehensive systemic phenotyping.
- Griffin JM, Baughan E, Rosenblum H, et al. Surveillance for disease progression of transthyretin amyloidosis after heart transplantation in the era of novel disease modifying therapies. J Heart Lung Transplant. Feb 2022;41(2):199–207. doi:https://doi.org/10.1016/j.healun.2021.10.007.
  - ○ This manuscript provides modern evidence supporting routine post-transplant surveillance and integration of disease-modifying therapy as key components of long-term management after heart transplantation for ATTR amyloidosis.

## Data Availability

No datasets were generated or analysed during the current study.
